# NOMADIC GERONTOLOGY: AGING AS A NOMAD IN MONGOLIA

**DOI:** 10.1093/geroni/igae098.0419

**Published:** 2024-12-31

**Authors:** Reuben Ng, Nicole Indran

**Affiliations:** National University of Singapore, Singapore, Singapore; National University of Singapore, Singapore, Singapore

## Abstract

Gerontologists have made remarkable strides in understanding the experiences of older adults in various contexts. Yet, amid this progress, some communities remain overlooked, such as older nomads in Mongolia. Notably, Mongolia is one of the last bastions of nomadism in the world. Today, many of the remaining nomads are individuals in their later years. This study aims to illuminate the lived experiences of older nomads in Mongolia. Specifically, we explore how they navigate the duality of being old and nomadic, particularly amid a changing social, political, economic and environmental setup. We interviewed a total of 32 Mongolian nomads whose average age was 65.3 years. Thematic analysis was employed to identify themes related to life as an older nomad. Three themes surfaced: ‘Navigating Health Challenges Arising from Aging Bodies and Climate Change’ (Theme 1), ‘Desire to Preserve a Disappearing Heritage’ (Theme 2) and ‘Experiencing Neglect and Discrimination’ (Theme 3). This study marks an important foray into research on older nomads, airing the voice of a community that is not only under-represented, but also fast vanishing. In today’s context, their nomadic ways stand as an in bulwark against the tide of modernization. Our findings make a compelling case for continued research and policy design to enhance the quality of life of older nomads, even as their cultural heritage is safeguarded.

